# Supplementary material for: Interface condition for the Darcy velocity at the water-oil flood front in the porous medium
Source: PLoS One. 2017 May 23;12(5):e0177187. doi: 10.1371/journal.pone.0177187 (PMC5441608; doi:10.1371/journal.pone.0177187)
Supplement: S1 File — (ZIP) [file pone.0177187.s001.zip › cases of JVCM/cases.pdf]

Solve the Buckley Leverete Equaiton
Relative Permeability designed by using Corey model.

Time : t=3day;

restart;

relatvie perm :

$Swn := Sw \rightarrow \frac{Sw}{1 - Swi - Sorw}$; $Krow := Sw \rightarrow (1 - Swn(Sw))^{No}$; $Krw := Sw \rightarrow K0rw \cdot Swn(Sw)^{Nw}$;

$$Sw \rightarrow \frac{Sw}{1 - Swi - Sorw}$$

$$Sw \rightarrow (1 - Swn(Sw))^{No}$$

$$Sw \rightarrow K0rw\ Swn(Sw)^{Nw} \qquad \textbf{(1.1.1)}$$

$K0rw := 0.7$; $No := 3$; $Nw := 2$; $Swi := 0$; $Sorw := 0$;

$$0.7$$

$$3$$

$$2$$

$$0$$

$$0 \qquad \textbf{(1.1.2)}$$

$fw := Sw \rightarrow \frac{\frac{Krw(Sw)}{\mu w}}{\frac{Krw(Sw)}{\mu w} + \frac{Krow(Sw)}{\mu o}}$; $plot([Krw(Sw),\ Krow(Sw)],\ Sw = 1..0)$; $\mu w := 0.5$;
$\mu o := 20$;

$$Sw \rightarrow \frac{Krw(Sw)}{\mu w \left(\frac{Krw(Sw)}{\mu w} + \frac{Krow(Sw)}{\mu o}\right)}$$

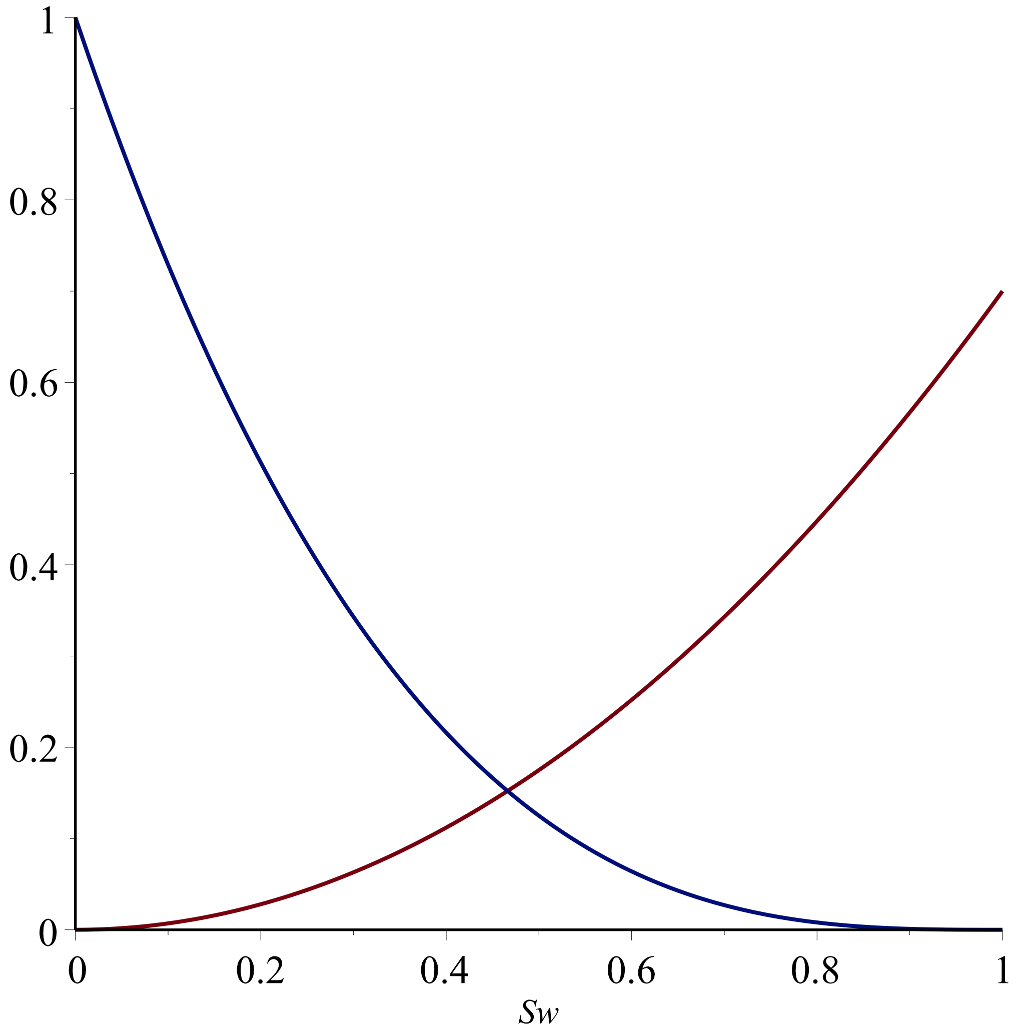

0.5

20 **(1.1.3)**

```
plot(fw(Sw), Sw = 0..1);
```

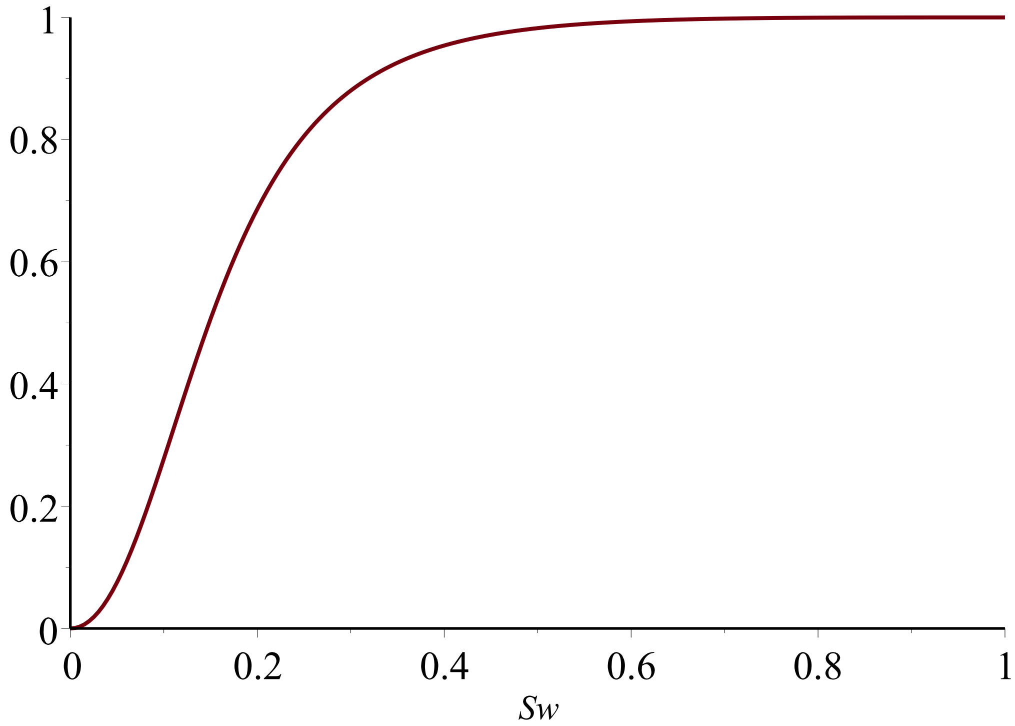

$dfw1 := diff(fw(Sw), Sw);\ dfw2 := diff(fw(Sw), Sw\$2);\ dfw1 := unapply(dfw1, Sw);\ dfw2 := unapply(dfw2, Sw);\ plot([fw(Sw), dfw1(Sw)], Sw = 0..1)$

$$\frac{2.800000000\ Sw}{1.400000000\ Sw^2 + \frac{1}{20}\ (1 - Sw)^3} - \frac{1.400000000\ Sw^2 \left(2.800000000\ Sw - \frac{3}{20}\ (1 - Sw)^2\right)}{\left(1.400000000\ Sw^2 + \frac{1}{20}\ (1 - Sw)^3\right)^2}$$

$$\frac{2.800000000}{1.400000000\ Sw^2 + \frac{1}{20}\ (1 - Sw)^3} - \frac{5.600000000\ Sw \left(2.800000000\ Sw - \frac{3}{20}\ (1 - Sw)^2\right)}{\left(1.400000000\ Sw^2 + \frac{1}{20}\ (1 - Sw)^3\right)^2} + \frac{2.800000000\ Sw^2 \left(2.800000000\ Sw - \frac{3}{20}\ (1 - Sw)^2\right)^2}{\left(1.400000000\ Sw^2 + \frac{1}{20}\ (1 - Sw)^3\right)^3}$$

$$-\frac{1.400000000\ Sw^2\left(3.100000000-\frac{3}{10}\ Sw\right)}{\left(1.400000000\ Sw^2+\frac{1}{20}\ (1-Sw)^3\right)^2}$$

$$Sw \rightarrow \frac{2.800000000\ Sw}{1.400000000\ Sw^2+\frac{1}{20}\ (1-Sw)^3}$$

$$-\frac{1.400000000\ Sw^2\left(2.800000000\ Sw-\frac{3}{20}\ (1-Sw)^2\right)}{\left(1.400000000\ Sw^2+\frac{1}{20}\ (1-Sw)^3\right)^2}$$

$$Sw \rightarrow \frac{2.800000000}{1.400000000\ Sw^2+\frac{1}{20}\ (1-Sw)^3}$$

$$-\frac{5.600000000\ Sw\left(2.800000000\ Sw-\frac{3}{20}\ (1-Sw)^2\right)}{\left(1.400000000\ Sw^2+\frac{1}{20}\ (1-Sw)^3\right)^2}$$

$$+\frac{2.800000000\ Sw^2\left(2.800000000\ Sw-\frac{3}{20}\ (1-Sw)^2\right)^2}{\left(1.400000000\ Sw^2+\frac{1}{20}\ (1-Sw)^3\right)^3}$$

$$-\frac{1.400000000\ Sw^2\left(3.100000000-\frac{3}{10}\ Sw\right)}{\left(1.400000000\ Sw^2+\frac{1}{20}\ (1-Sw)^3\right)^2}$$

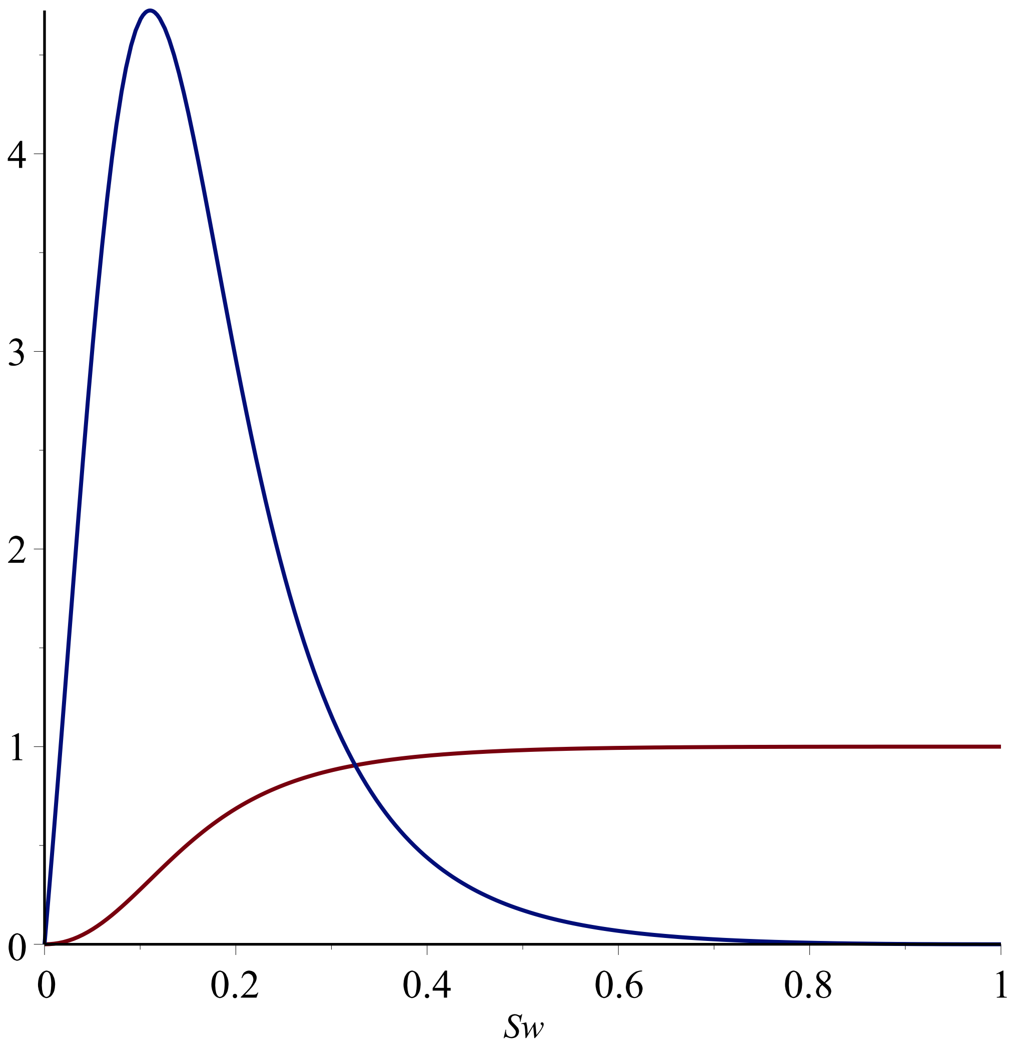

$$eq := \frac{fw(x) - 0}{x - 0} = diff(fw(x),\ x)$$

$$\frac{1.400000000\ x}{1.400000000\ x^2 + \frac{1}{20}\ (1-x)^3} = \frac{2.800000000\ x}{1.400000000\ x^2 + \frac{1}{20}\ (1-x)^3} - \frac{1.400000000\ x^2 \left(2.800000000\ x - \frac{3}{20}\ (1-x)^2\right)}{\left(1.400000000\ x^2 + \frac{1}{20}\ (1-x)^3\right)^2} \tag{1.1.4}$$

$solve(eq,\ x)$

$$0.,\ 0.1806612423,\ 15.49791828,\ -0.1785795177 \tag{1.1.5}$$

$Swf := 0.1806612423;\ t := 3.1;\ K := 500;\ A := 1;\ q := 1;\ \phi := 0.15;$

0.1806612423

3.1

500

1

1

0.15 **(1.1.6)**

for *Sw* **from** *Swf* **by** 0.01 **to** 1 **do** $print\left(\frac{dfw1(\mathrm{Sw})}{\mathrm{A}\cdot\phi}\cdot\mathrm{q}\cdot\mathrm{t}\right)$ **end do**;

71.41316076

66.06803378

60.84147987

55.81724741

51.05174978

46.57940312

42.41754084

38.57068295

35.03408995

31.79661980

28.84295833

26.15531448

23.71467543

21.50170958

19.49739643

17.68344668

16.04256636

14.55860690

13.21663300

12.00293288

10.90499067

9.911432446

9.011958394

8.197266118

7.458971092

6.789528262

6.182154726

5.630757766

5.129867166

4.674572916

4.260467786

3.883595330

3.540403010
3.227699624
2.942617505
2.682577560
2.445260015
2.228575595
2.030642166
1.849762994
1.684406659
1.533190395
1.394864427
1.268298391
1.152468999
1.046448855
0.9493975680
0.8605520432
0.7792193272
0.7047695000
0.6366296192
0.5742777072
0.5172385960
0.4650786780
0.4174022900
0.3738480752
0.3340858012
0.2978133420
0.2647542807
0.2346553267
0.2072843313
0.1824283660
0.1598920480
0.1394959080
0.1210749640
0.1044775433
0.08956412532
0.07620620468

$$0.06428544400$$
$$0.05369272332$$
$$0.04432749932$$
$$0.03609683400$$
$$0.02891514667$$
$$0.02270313933$$
$$0.01738783800$$
$$0.01290162133$$
$$0.009181786668$$
$$0.006170612000$$
$$0.003814446668$$
$$0.002063690667$$
$$0.0008724226668$$
$$0.0001980280000 \qquad (1.1.7)$$

$Swf := 0.1806612423;\ t := 3.1;\ K := 500;\ A := 1;\ q := 1;\ \phi := 0.15;$

$$0.1806612423$$
$$3.1$$
$$500$$
$$1$$
$$1$$
$$0.15 \qquad (1.1.8)$$

$$P := y \rightarrow 40 - \frac{q \cdot q \cdot t}{0.086400 \cdot A \cdot A \cdot \phi \cdot K} \cdot int\left(\frac{\mathrm{dfw2}(x)}{\left(\frac{Krow(x)}{\mu o} + \frac{Krw(x)}{\mu w}\right)}, x = 1 .. y\right);$$

$$y \rightarrow 40 + \frac{(-1)\ q\ q\ t \left(\int_1^y \frac{dfw2(x)}{\frac{Krow(x)}{\mu o} + \frac{Krw(x)}{\mu w}}\, \mathrm{d}x\right)}{0.086400\ A\ A\ \phi\ K}$$

for *Sw* **from** *Swf* **by** 0.01 **to** 1 **do** *print*(P(*Sw*)) **end do**;

$$25.98251505$$
$$27.62680353$$
$$29.14681314$$
$$30.52730509$$
$$31.76395471$$
$$32.85990387$$

33.82303892
34.66394966
35.39447018
36.02668526
36.57229151
37.04221739
37.44642517
37.79383562
38.09233178
38.34881067
38.56926140
38.75885514
38.92203782
39.06261986
39.18385961
39.28853906
39.37903115
39.45735892
39.52524689
39.58416542
39.63536870
39.67992723
39.71875553
39.75263557
39.78223674
39.80813268
39.83081567
39.85070861
39.86817538
39.88352943
39.89704121
39.90894433
39.91944093
39.92870609
39.93689175
39.94412987

39.95053529
39.95620802
39.96123533
39.96569344
39.96964902
39.97316046
39.97627895
39.97904944
39.98151146
39.98369978
39.98564503
39.98737425
39.98891132
39.99027733
39.99149095
39.99256873
39.99352534
39.99437381
39.99512569
39.99579128
39.99637973
39.99689921
39.99735697
39.99775951
39.99811263
39.99842149
39.99869073
39.99892449
39.99912647
39.99930000
39.99944806
39.99957332
39.99967817
39.99976477
39.99983505
39.99989075

39.99993342
39.99996448
39.99998519
39.99999668 **(1.1.10)**

Diff(P(x),x)

$x0 := 71.41316076$

71.41316076 **(1.2.1)**

$$v_o + v_w = 0.086400 * A \cdot K \cdot \left(\frac{k_{ro}}{\mu_o} + \frac{k_{rw}}{\mu_w} \right) \cdot dPx = qconst$$

false **(1.2.2)**

$$dPx := Sw \rightarrow \frac{q}{0.086400 * A \cdot K \cdot \left(\frac{Krow(Sw)}{\mu o} + \frac{Krw(Sw)}{\mu w} \right)}$$

$$Sw \rightarrow \frac{q}{0.086400 \; A \; K \left(\frac{Krw(Sw)}{\mu w} + \frac{Krow(Sw)}{\mu o} \right)}$$ **(1.2.3)**

for *Sw* **from** *Swf* **by** 0.01 **to** 1 **do** *print*(-*dPx*(*Sw*)) **end do**;

-0.3162503725
-0.2990739226
-0.2826133566
-0.2669312232
-0.2520617057
-0.2380165958
-0.2247905310
-0.2123654313
-0.2007141488
-0.1898034013
-0.1795960761
-0.1700530016
-0.1611342818
-0.1528002754
-0.1450122946
-0.1377330857
-0.1309271419
-0.1245608871

-0.1186027681
-0.1130232750
-0.1077949148
-0.1028921497
-0.09829131214
-0.09397050840
-0.08990951117
-0.08608965243
-0.08249371386
-0.07910582209
-0.07591134606
-0.07289680114
-0.07004975785
-0.06735875696
-0.06481323045
-0.06240342812
-0.06012035046
-0.05795568600
-0.05590175474
-0.05395145537
-0.05209821781
-0.05033595900
-0.04865904310
-0.04706224485
-0.04554071609
-0.04408995527
-0.04270577957
-0.04138429948
-0.04012189547
-0.03891519677
-0.03776106193
-0.03665656111
-0.03559895970
-0.03458570358
-0.03361440536
-0.03268283200

-0.03178889325
-0.03093063122
-0.03010621072
-0.02931391034
-0.02855211437
-0.02781930525
-0.02711405671
-0.02643502737
-0.02578095493
-0.02515065071
-0.02454299469
-0.02395693092
-0.02339146321
-0.02284565123
-0.02231860684
-0.02180949076
-0.02131750931
-0.02084191172
-0.02038198722
-0.01993706271
-0.01950650025
-0.01908969508
-0.01868607356
-0.01829509122
-0.01791623107
-0.01754900201
-0.01719293723
-0.01684759289 **(1.2.4)**

(1.2.5)

Caculate the Pressure Field

$$\frac{dP}{dx} = \frac{qconst}{0.086400 * A * K * \left(\frac{k_{ro}}{\mu_o} + \frac{k_{rw}}{\mu_w}\right)}; \; dx = \frac{q(t)\Delta t}{A\varphi} f_w'' \, dS$$

$$dP = \frac{\frac{q(t) * \Delta t}{A \cdot \varphi} f_w'' \cdot dS}{0.086400 * A * K * \left(\frac{k_{ro}}{\mu_o} + \frac{k_{rw}}{\mu_w}\right)}$$

$$P := y \rightarrow 40 - \frac{q \cdot q \cdot t}{0.086400 * A \cdot A \cdot \phi * K} \cdot int\left(\frac{dfw2(x)}{\left(\frac{Krow(x)}{\mu o} + \frac{Krw(x)}{\mu w}\right)}, x = 1 .. y\right);$$

$$dp2 := \frac{1}{0.086400 * A * K * \left(\frac{1}{\mu o}\right)}; \; p0 := 28.57445157;$$

0.4629629628

28.57445157 **(1.3.1)**

p2:=$p0 - dp2 \cdot (100 - x0)$

14.27329899 **(1.3.2)**

$dp2 \cdot (100 - 62.88758843)$

17.18167202 **(1.3.3)**

Caculate Krw and Krow Field：

for *Sw* **from** *Swf* **by** 0.01 **to** 1 **do** *print*(Krw(*Sw*)) **end do**;

0.02284693913
0.02544619652
0.02818545391
0.03106471131
0.03408396870
0.03724322609
0.04054248348
0.04398174087
0.04756099827
0.05128025566
0.05513951305
0.05913877045
0.06327802783
0.06755728523
0.07197654261
0.07653580004

0.08123505740
0.08607431483
0.09105357219
0.09617282955
0.1014320870
0.1068313443
0.1123706018
0.1180498591
0.1238691166
0.1298283739
0.1359276314
0.1421668887
0.1485461461
0.1550654035
0.1617246609
0.1685239183
0.1754631756
0.1825424331
0.1897616904
0.1971209479
0.2046202052
0.2122594627
0.2200387200
0.2279579774
0.2360172348
0.2442164922
0.2525557496
0.2610350070
0.2696542644
0.2784135218
0.2873127792
0.2963520365
0.3055312940
0.3148505513
0.3243098088
0.3339090661

0.3436483236

0.3535275809

0.3635468383

0.3737060957

0.3840053531

0.3944446105

0.4050238678

0.4157431253

0.4266023826

0.4376016401

0.4487408974

0.4600201549

0.4714394122

0.4829986696

0.4946979270

0.5065371844

0.5185164418

0.5306356992

0.5428949566

0.5552942140

0.5678334714

0.5805127287

0.5933319862

0.6062912435

0.6193905010

0.6326297583

0.6460090158

0.6595282731

0.6731875305

0.6869867879 **(1.4.1)**

```
for Sw from Swf by 0.01 to 1 do print(Krow(Sw)) end do;
```

0.5500352174

0.5107314639

0.4733461234

0.4378311960

0.4041386815

0.3722205801
0.3420288917
0.3135156164
0.2866327540
0.2613323047
0.2375662684
0.2152866451
0.1944454348
0.1749946375
0.1568862533
0.1400722821
0.1245047238
0.1101355787
0.09691684649
0.08480052734
0.07373862121
0.06368312810
0.05458604800
0.04639938093
0.03907512687
0.03256528583
0.02682185781
0.02179684280
0.01744224082
0.01371005186
0.01055227591
0.007920912980
0.005767963070
0.004045426178
0.002705302305
0.001699591450
0.0009802936143
0.0004994087966
0.0002089369975
0.00006087821680
0.000007232454600 **(1.4.2)**
